# HIV-1 Tat-Mediated Induction of CCL5 in Astrocytes Involves NF-κB, AP-1, C/EBPα and C/EBPγ Transcription Factors and JAK, PI3K/Akt and p38 MAPK Signaling Pathways

**DOI:** 10.1371/journal.pone.0078855

**Published:** 2013-11-11

**Authors:** Anantha R. Nookala, Ankit Shah, Richard J. Noel, Anil Kumar

**Affiliations:** 1 Division of Pharmacology and Toxicology, UMKC-School of Pharmacy, Kansas City, Missouri, United States of America; 2 Department of Biochemistry, Ponce School of Medicine and Health Sciences, Ponce, Puerto Rico, United States of America; Temple University School of Medicine, United States of America

## Abstract

The incidence of HIV-associated neurological disorders (HAND) has increased during recent years even though the highly active antiretroviral therapy (HAART) has significantly curtailed the virus replication and increased the life expectancy among HIV-1 infected individuals. These neurological deficits have been attributed to HIV proteins including HIV-1 Tat. HIV-1 Tat is known to up-regulate CCL5 expression in mouse astrocytes, but the mechanism of up-regulation is not known. The present study was undertaken with the objective of determining the mechanism(s) underlying HIV-1 Tat-mediated expression of CCL5 in astrocytes. SVGA astrocytes were transiently transfected with a plasmid encoding Tat, and expression of CCL5 was studied at the mRNA and protein levels using real time RT-PCR and multiplex cytokine bead array, respectively. HIV-1 Tat showed a time-dependent increase in the CCL5 expression with peak mRNA and protein levels, observed at 1 h and 48 h post-transfection, respectively. In order to explore the mechanism(s), pharmacological inhibitors and siRNA against different pathway(s) were used. Pre-treatment with SC514 (NF-κB inhibitor), LY294002 (PI3K inhibitor), AG490 (JAK2 inhibitor) and Janex-1 (JAK3 inhibitor) showed partial reduction of the Tat-mediated induction of CCL5 suggesting involvement of JAK, PI3K/Akt and NF-κB in CCL5 expression. These results were further confirmed by knockdown of the respective genes using siRNA. Furthermore, p38 MAPK was found to be involved since the knockdown of p38δ but not other isoforms showed partial reduction in CCL5 induction. This was further confirmed at transcriptional level that AP-1, C/EBPα and C/EBPγ were involved in CCL5 up-regulation.

## Introduction

Human immunodeficiency virus-1 (HIV-1) enters the brain through blood brain barrier (BBB) early after the infection [1]. Prolonged infection of central nervous system (CNS) further leads to various neurological complications including HIV-associated dementia (HAD). After the advent of HAART, the incidence of HAD has reduced; however, due to the prolonged life-span, neurological deficits are known to result into a collection of minor cognitive impairments known as HAND [2]. The neurotoxicity of HIV-1 has been attributed to the virus itself or the viral proteins shed after the infection *via* several mechanisms including production of cytokines/chemokines. In particular, presence of HIV-1 Tat has been reported in postmortem CNS tissue (hippocampus) of the HIV-1 infected patients, which underscores the significance of HIV-1 Tat in the HIV neuropathogenesis [3].

HIV-1 Trans-activator of transcription (HIV-1 Tat or Tat) is a functional protein that is produced very early during the HIV-1 virus replication. It binds to the Tat associated region on the viral RNA and increases the replication of the virus [4], [5]. Tat has been found to be toxic to the mice when injected into the cerebroventricular region [6], [7]. The neurotoxicity of Tat is attributed to various mechanisms such as, over excitation of the neurons *via* N-methyl-D-aspartate receptor [8], [9], [10], [11] increasing intracellular calcium levels [12], [13], [14] and disrupting the normal function of electron transport chain [15]. In addition, Tat induces a bystander effect on neurons by producing neurotoxic substances such as pro-inflammatory cytokines/chemokines [16], [17], nitric oxide synthase [18], [19] and quinolinic acid from the adjacent astrocytes and microglia [20]. Furthermore, Tat also affects the integrity of the BBB by altering the tight junction proteins [21], by inducing oxidative stress [22], [23], [24] and apoptosis [25] in brain microvascular endothelial cells.

Astrocytes are the most abundant cells of the CNS and occupy more than 50% of the brain volume. They play a vital role in CNS homeostasis by performing various functions such as promoting the release of various neurotrophic factors, increasing the number of synapses and maintaining synaptic plasticity and also promoting the uptake of excitatory neurotransmitters including glutamate, released by the neurons [26]. Furthermore, they function as immune cells in the CNS by releasing myriad of cytokines/chemokines such as interleukins, (IL-1β, IL-6, IL-8), Interferons (IFNs) and Chemokine ligands (CCLs) including CCL5 [27]. CCL5 [CC-chemokine ligand 5; also called RANTES (Regulated upon activation, normal T-cell expressed, and secreted)] is a β-chemokine that plays an important role in inflammation by acting on C-C chemokine receptor type 5 (CCR5), which is a G-protein coupled receptor. Furthermore, during viral infection, it directs the lymphocytes and monocytes to the site of inflammation [28]. Increased levels of CCL5 has been implicated in the pathology of various diseases such as Alzheimer’s disease [29], Parkinson’s disease [30], Multiple sclerosis [31], asthma [32] and HIV-1 infection [33]. Previous studies have shown CCL5-mediated increase in the replication of T-tropic strains of HIV-1 *via* Gi protein-mediated transduction [34] and also that HIV-1 Tat can induce CCL5 production in astrocytes [35].

In the present study, we sought to determine the signaling pathway(s) underlying the HIV-1 Tat-mediated CCL5 production in astrocytes. First, we tested whether Tat induced CCL5 in a time-dependent manner. We then studied the possible involvement of nuclear factor κB (NF-κB) and other transcription factors as well as PI3K/Akt, p38/JNK MAPKs and Janus Kinases (JAK1/2/3) as upstream signaling pathways in CCL5 production.

## Materials and Methods

### Cell Culture and Reagents

SVGA cells (Astroglial cells modified from simian virus 40 (SV40)-transformed human glial cells (SVG)) were a generous gift from Dr. Avindra Nath which were originally developed by Major et al [36]. The cells were grown in Dulbecco’s Modified Eagle Medium (DMEM) supplemented with 10% heat-inactivated fetal bovine serum, 1% non-essential amino acids, 1% sodium bicarbonate, 1% L-glutamine and 50 µg/ml of gentamycin. The cells were maintained in an incubator at 37°C and humidified air with 5% CO_2_. NF-κB inhibitor (SC514), p38 inhibitor (SB203580), JNK inhibitor (SP600125), PI3K inhibitor (LY294002), JAK1 inhibitor (Picetannol), JAK2 inhibitor (AG490) and JAK3 inhibitor (Janex-1) were obtained from Cayman chemical (Ann Arbor, MI, USA). Pre-designed siRNA against p50, p65, p38 (α/β/γ/δ) were purchased from Ambion Inc (Carlsbad, CA,USA) and siRNA against C/EBPα, C/EBPγ, AP-1, Akt (1/2/3) and JAK (1/2/3) were purchased from Thermo Fisher Scientific (PA,USA).

### Transfection of Astrocytes with HIV-1 Tat Plasmid and siRNA

A plasmid encoding HIV-1 Tat, constructed by Dr. E Verdin, Gladstone Institute, UCSF, was obtained from NIH AIDS Reagent Program (Catalog # 10453) [37]. SVGA cells were transiently transfected with plasmid encoding HIV-1 Tat using Lipofectamine 2000™ (Life technologies, NY, USA) as described previously [38]. Briefly, the cells were seeded in 6-well plate at a density of 0.7×10^6^ cells/well overnight before transfection. Next morning the medium was removed, and the cells were washed twice with PBS. The transfection mixture containing Opti-MEM, Lipofectamine 2000 and 0.3 µg of plasmid encoding Tat were added to the cells with 1 ml of serum-free medium. Transfection was terminated after 5 h and the transfection medium was replaced with complete DMEM. The cells and supernatant were collected at different times.

Experiments with pharmacological antagonists were performed by pretreating the cells 1 h prior to transfection with the plasmid coding for HIV-1 Tat. The doses of the antagonists were determined based on their IC50 values and dosage used by others [39], [40], [41], [42], [43], [44], [45]. However, in some cases previously used concentration caused significant death in SVGA cells. Therefore, we further optimized dose for those inhibitors (data not shown).

For experiments with small interfering RNA (siRNA), 0.55×10^6^ cells/well were seeded in a 6-well plate and were allowed to adhere overnight before transfection with 50 nM siRNA. Briefly, complete media was removed from the plates and cells were washed twice with PBS before addition of serum free medium. The transfection mixture containing siRNA, Opti-MEM and Lipofectamine were added into the wells. After 24 h, the transfection mixture was replaced with complete media and the cells were allowed to grow for 10 h before re-seeding into a 12 well plate. The transfection with siRNA was performed for 24 h as opposed to 5 h (for Tat plasmid) in order to ensure the maximum knockdown of the target as reported previously [46], [47]. These cells were then transiently transfected with the HIV-1 Tat plasmid for 5 h and the cells were harvested at 6 h for mRNA and at 48 h for protein.

### Quantitative Real Time RT-PCR

Total RNA was isolated from the cells using Qiagen RNeasy kit (QIAGEN, Valencia, CA) as per the manufacturer’s protocol. The CCL5 mRNA expression levels were measured using real-time reverse transcription polymerase chain reaction (RT-PCR). Briefly, 150 ng of RNA was reverse transcribed and amplified using the primers and PCR conditions as mentioned previously [38]. The expression levels of CCL5 were calculated by 2^−ΔΔCt^ method using hypoxanthine phosphoribosyltransferase (HPRT) as an internal housekeeping gene.

### Multiplex Cytokine Assay

The protein levels of CCL5 were measured using multiplex cytokine assay kit (Bio-Rad, CA, USA) as per the manufacturer’s protocol. Briefly, cell culture supernatants were collected from the plates at 6 h, 12 h, 24 h, 48 h, 72 h, 96 h post-transfection followed by centrifugation twice at 1000 g for 5 min. 50 µl of the samples and standards were mixed with magnetic beads and incubated on a shaker at room temperature for 30 min. The beads were washed and 25 µl of detection antibody was added to each well followed by incubation for 30 min. The samples were washed and incubated with 50 µl of streptavidin-PE conjugate for 10 min. Finally 125 µl of the assay buffer was added and the samples were analyzed using Biorad Bioplex HTS (Bio-Rad, CA, USA). The concentration of CCL5 was determined with the Bio-plex manager 5 using 5-PL statistics.

### Immunocytochemistry

SVGA cells were seeded at 0.6×10^6^ cells/well in a 6-well plate on glass cover slips in each well. The cells were allowed to adhere overnight followed by transfection with the HIV-1 Tat plasmid for 12 h. 6 h prior to the termination, 1 mg/ml Golgi-stop™ (BD Biosciences, CA, USA) solution was added into each well in order to prevent the release of CCL5. After termination, the cells were fixed by adding 1∶1 ice cold methanol and acetone solution and kept at −20°C for 20 min. The wells were air dried and the cover slips were incubated for 10 min in PBST (0.1% Triton-X in PBS). This was followed by 3 washes with PBS and blocking with 1% bovine serum albumin in PBST for 30 min. The cells were washed and further incubated for overnight with the mixture of antibodies for mouse anti-glial fibrillary acidic protein (anti-GFAP) (1∶1000) and rabbit anti-CCL5 (1∶500). After 3 washes with PBST, the secondary antibodies (Alexafluor 555 labeled Anti-mouse IgG and Alexafluor 488 labeled anti-rabbit IgG) were added at a dilution of 1∶1000 and the cover slips were incubated in dark for 1 h. The cover slips were gently taken out from the wells and washed with PBST before being transferred onto a glass slide containing the mounting medium with 4′,6-diamidino-2-phenylindole (DAPI) (Vector Laboratories, Burlingame, CA). The images were obtained using inverted confocal microscope, Leica TCS SP5 II (Leica Microsystems, Wetzler, Germany). The intensity of CCL5 was calculated using imageJ software and GFAP was used as a house keeping gene to normalize the intensity values.

### Statistical Analysis

Student’s t-test was used to calculate the statistical significance for time kinetics experiments. One-way ANOVA was used to calculate the statistical significance for all experiments involving the use of inhibitors and siRNA. All the experiments were performed in triplicates and the results are represented by the mean ± standard error (SE) of at least three individual experiments. p-value of ≤0.05 was considered to be statistically significant.

## Results

### HIV-1 Tat Induced the Expression of CCL5 in a Time-dependent Manner

**Figure 1:**
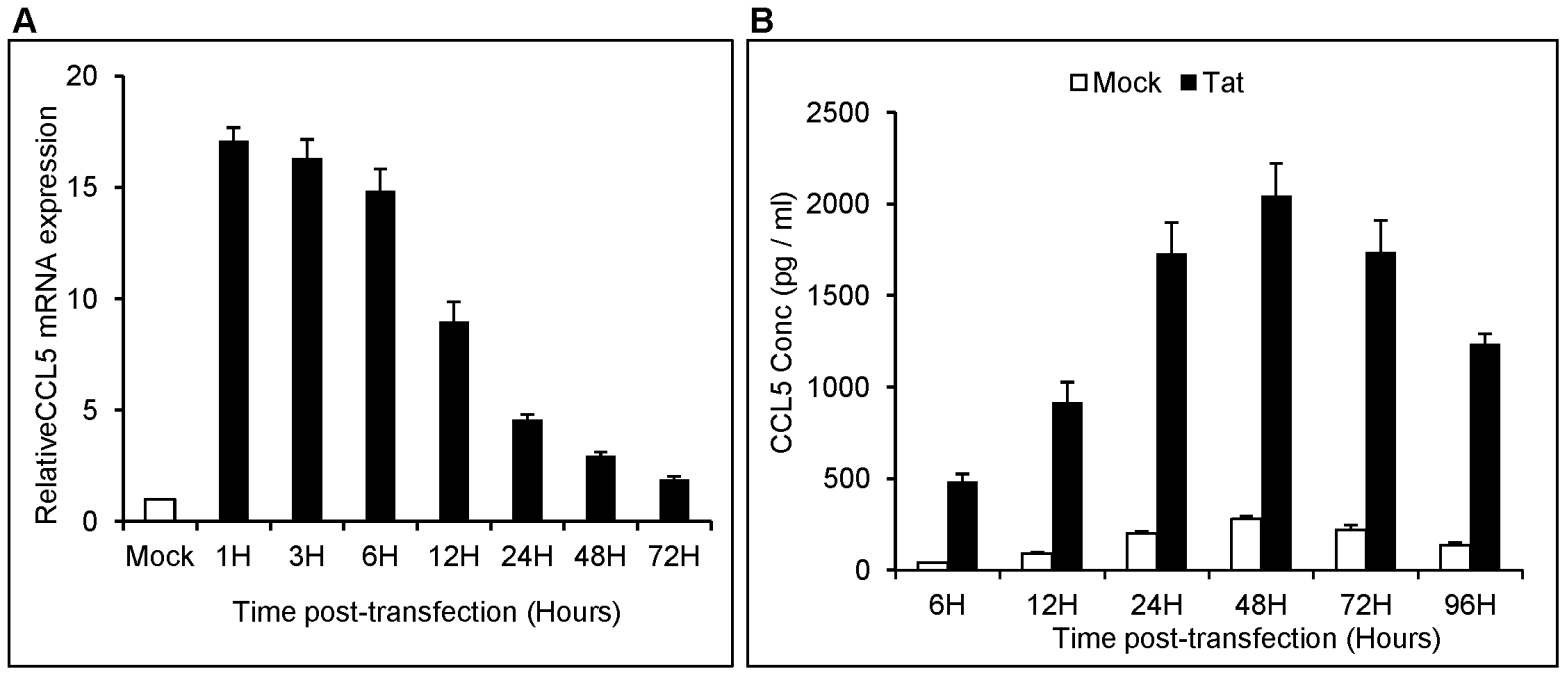
HIV-1 Tat induces CCL5 in SVGA astrocytes in a time dependent manner. 7×10^5^ SVGA astrocytes were transiently transfected with plasmid encoding HIV-1 Tat for 5 h using Lipofectamine 2000™. (A) The cells were harvested at 1 h, 3 h, 6 h, 12 h, 24 h, 48 h and 72 h and expression levels of the CCL5 mRNA were determined by RT-PCR. Data presented in the figure is relative to the mock-transfected controls. (B) CCL5 protein concentrations in the supernatants were measured at 6 h, 12 h, 24 h, 48 h, 72 h and 96 h by multiplex cytokine assay. Each experiment was performed in triplicate and each bar in the figure represents the mean ± SE of three individual experiments. Student’s t-test was employed to calculate the significance and p-value was found to be ≤0.0001 in all the cases.

In view of the findings that elevated CCL5 has been detected in the CSF of HIV-1 infected individuals suffering from HAD and that HIV-1 Tat induces CCL5 production in astrocytes [48], [49], we sought to investigate the underlying mechanism(s) responsible for HIV-1 Tat-mediated CCL5 expression in astrocytes. For the purpose we used a transfection model where astrocytes are transiently transfected with HIV-1 Tat plasmid with the transfection efficiency of 55–70% (data not shown). We observed elevated CCL5 mRNA level within 1 h of transfection (17.09±0.59 fold), which gradually declined in a time-dependent manner over 72 h observation period (Fig. 1A). Similarly, the expressions at protein levels were measured in cell culture supernatants at various time intervals (6 h, 12 h, 24 h, 48 h, 72 h and 96 h) (Fig. 1B). The protein levels of CCL5 showed significant increase as early as 6 h (0.48±0.04 ng/ml *vs* 0.04±0.001 ng/ml in control). The peak CCL5 expression was observed at 48 h post-transfection (2.04±0.17 ng/ml compared to 0.27±0.01 ng/ml in controls) followed by time-dependent decrease over 96 h observation period (Fig. 1B). These results indicate that HIV-1 Tat-mediated induction of CCL5 expression follows a time-dependent kinetics at both mRNA as well as protein levels.

**Figure 2:**
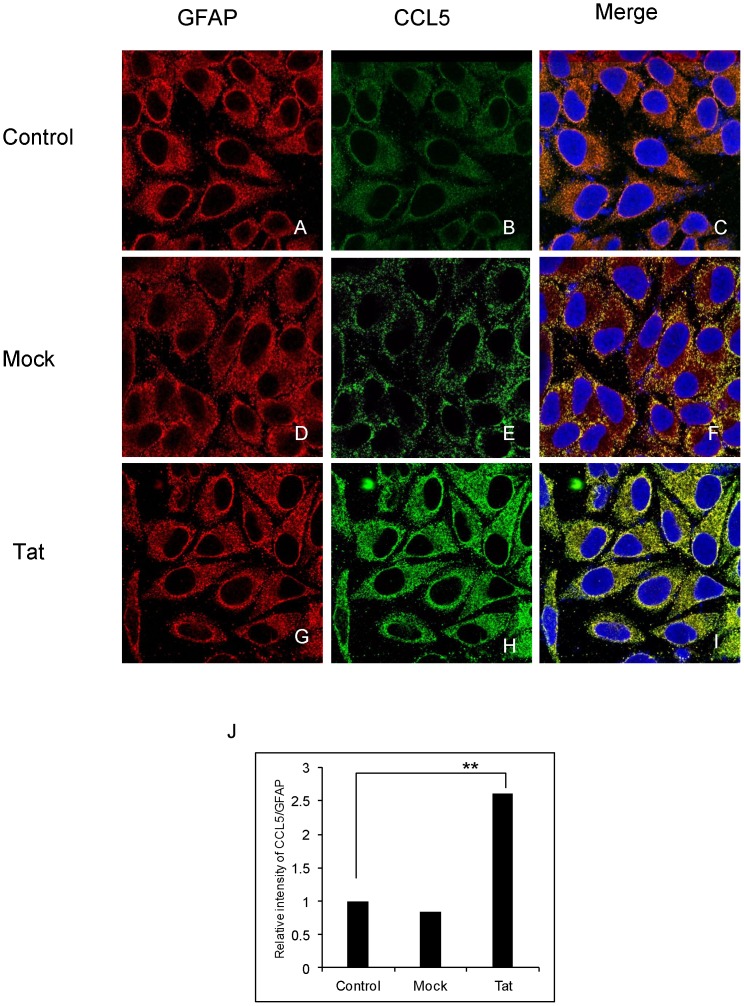
Immunocytochemistry of CCL5 induced by HIV-1 Tat in astrocytes. (A–I) SVGA astrocytes were grown on a cover slip before transfecting with HIV-1 Tat plasmid. Untransfected control (A–C) and mock-transfected cells (D–F) were used to compare the up-regulation of CCL5 in cells transfected with HIV-1 Tat (G–I). The cells were stained with the primary antibodies against CCL5 and GFAP and secondary antibody labeled with Alexafluor 555 (GFAP) and Alexafluor 488 (CCL5). Finally the cover slips were mounted on medium containing DAPI to stain the nucleus. The merge panels represent the co-localization of CCL5 with GFAP. The images were captured using inverted confocal microscope, Leica TCS SP5 II. (J) The intensity of CCL5 over GFAP was calculated using imageJ software. Student’s t-test was employed to calculate the significance and ** denotes the p-value ≤0.01.

In order to further confirm the findings observed at mRNA and protein levels, we employed immunocytochemistry on the HIV-1 Tat-transfected astrocytes to visualize the production of CCL5. The astrocytes showed strong GFAP (Red) staining with no significant difference among control, mock and HIV-1 Tat-transfected cells (Figures 2A, D and G, respectively). On the other hand control astrocytes showed basal level of CCL5 (green) staining (Fig. 2B) which slightly decreased in mock-transfected cells (Fig. 2E). However, as shown in Fig. 2H, the CCL5 signal was significantly stronger in astrocytes transfected with HIV-1 Tat plasmid. Figures 2 C, F and I show merged staining with nuclei (blue) in the center. The calculated relative intensity of CCL5 over GFAP for Tat-transfected astrocytes was 2.6-fold higher when compared to the untransfected control cells. The mock-transfected astrocytes showed a non-significant decrease in the intensities for CCL5/GFAP as compared to the untransfected cells (Fig. 2J).

### HIV-1 Tat-mediated Induction of CCL5 Involves NF-κB Pathway

**Figure 3:**
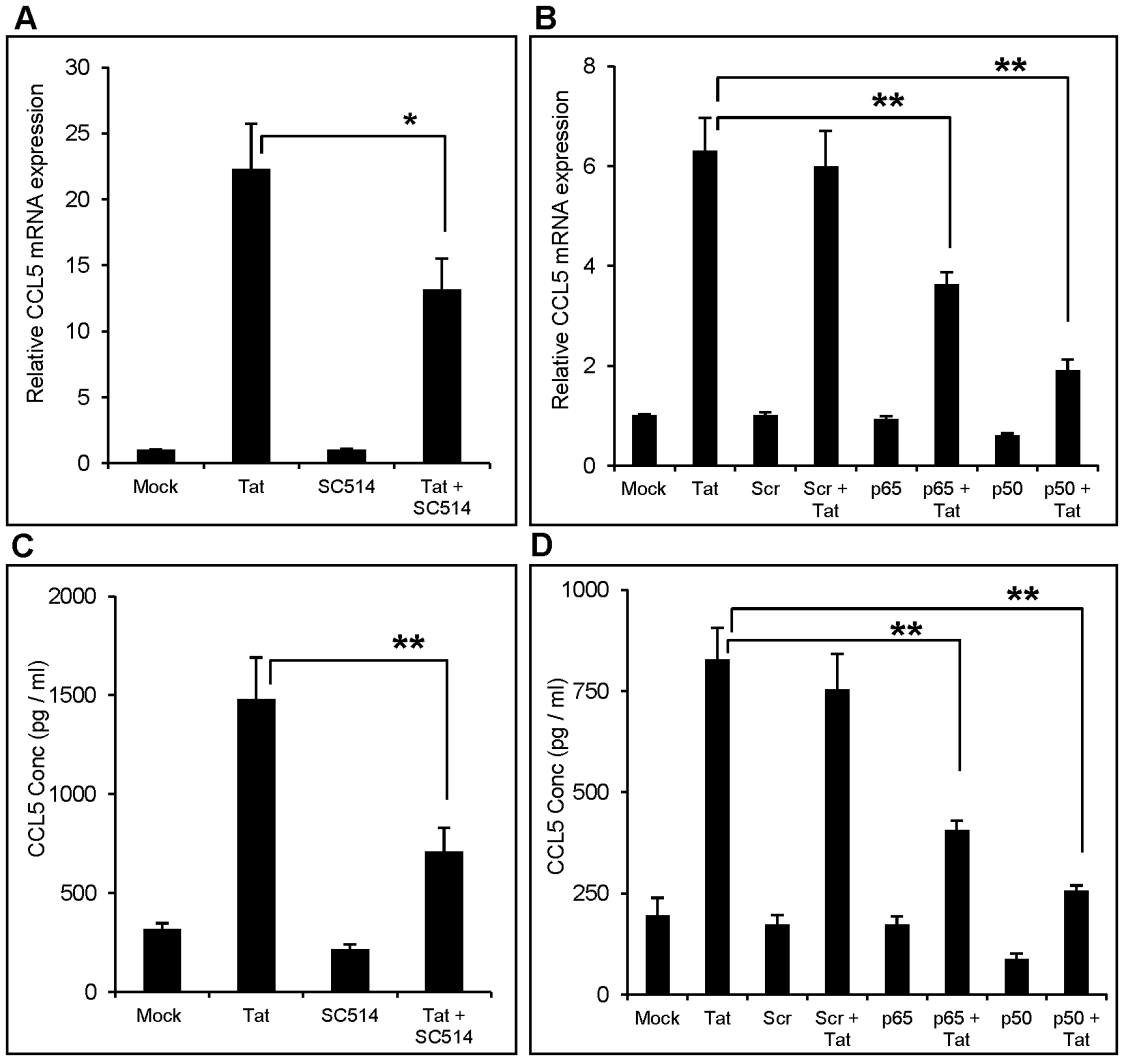
Involvement of NF-κB in HIV-1 Tat mediated production of CCL5 from astrocytes. (A, B) SVGA astrocytes were treated with 10 µM of NF-κB inhibitor (SC514) prior to transfection with plasmid encoding HIV-1 Tat. (C, D) For knockdown using siRNA, the cells were transfected with the siRNA followed by Tat transfection as mentioned in the Materials and Methods. The expression of CCL5 at the mRNA and protein levels were measured at 6 h and 48 h post-transfection by using RT-PCR (A,C) and multiplex cytokine assay (B, D), respectively. The values represented for mRNA are expressed relative to the mock-transfected controls. Each experiment was performed in triplicate and each bar in the figure represents the mean ± SE of at least three individual experiments. One-way ANOVA was used to perform the statistical analysis and ** denotes p-value ≤0.01 and * denotes p-value of ≤0.05.

NF-κB is a major transcriptional factor that plays an important role in the process of inflammation by regulating the expression of variety of cytokines and chemokines [50], [51]. Therefore, we sought to determine if NF-κB is involved in CCL5 production in astrocytes by HIV-1 Tat. The cells were pretreated with SC514, a specific inhibitor for NF-κB, 1 h prior to the transfection with Tat plasmid and the expression levels of CCL5 were determined 6 h and 48 h post-transfection for mRNA and protein, respectively. As shown in Fig. 3A (mRNA) and 3B (protein), SC514 decreased the expression of CCL5 by 46.6±14.2% and 47.7±11.9% at RNA and protein levels, respectively.

In order to confirm the results of pharmacological antagonists, we used siRNA to knock down specific genes, p50 (NF-κB1) and p65 (RelA), responsible for NF-κB activation. As expected, the p65 and p50 knockdown resulted in partial reduction of CCL5 expression at mRNA by 42.8±8.3% and 69.8±10.5% (Fig. 3C). Similar reductions were observed at the protein level, where p65 and p50 knockdown reduced CCL5 production by 48.9±6.07% and 68.9±4.86%, respectively (Fig. 3D).

### Involvement of p38 MAPK Pathway in Induction of CCL5 by HIV-1 Tat

**Figure 4:**
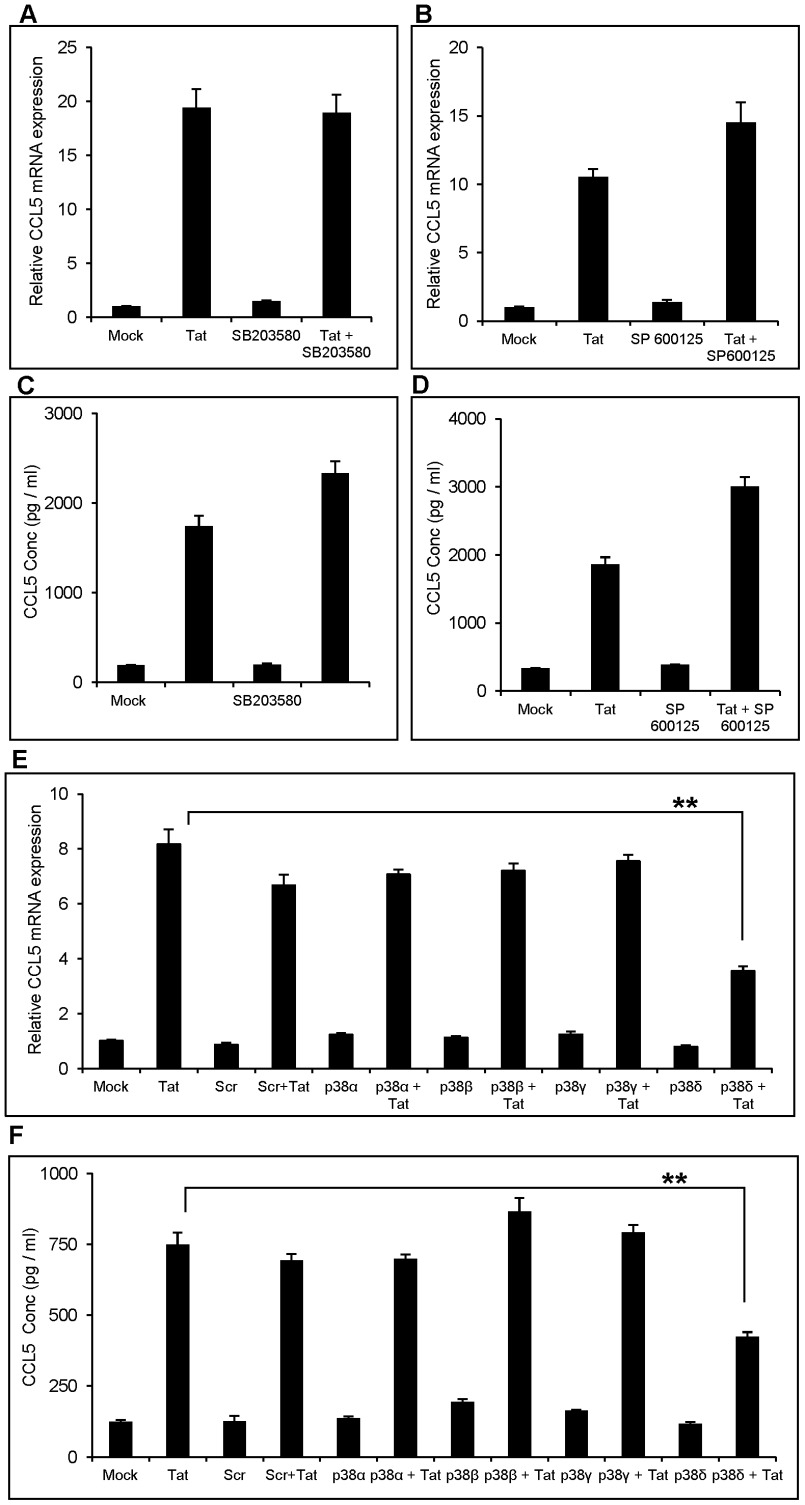
Inhibition of p38 MAPK reduces the induction of CCL5 by HIV-1 Tat. (A–D) SVGA astrocytes were pretreated with 10 µM of p-38 MAPK chemical inhibitor (SB203580) or JNK MAPK chemical inhibitor (SP600125) 1 h prior to the transfection with Tat plasmid. (E, F) For gene knockdown with siRNA, the cells were transfected with siRNA against p38α, p38β, p38γ or p38δ before transfecting with HIV-1 Tat plasmid as mentioned in the Materials and Methods. The expression of CCL5 at the mRNA and protein levels were measured at 6 h and 48 h post-transfection by using RT-PCR (A,C,E) and multiplex cytokine assay (B,D,F), respectively. The values represented for mRNA are expressed relative to the mock-transfected controls. Each experiment was performed in triplicate and each bar in the figure represents the mean ± SE of three individual experiments. One-way ANOVA was used to perform the statistical analysis and ** denotes p-value ≤0.01.

After determining the role of NF-κB, we wanted to explore the upstream signaling pathways involved in the activation of NF-κB. Various Mitogen-activated protein kinases (MAPKs) such as p38 MAPK, ERK MAPK and JNK MAPK are known to activate NF-κB as reported previously [52]. We, therefore, used specific pharmacological antagonists against p38 MAPK (SB203580) and JNK MAPK (SP600125). Neither of these inhibitors affected CCL5 expression at mRNA and protein levels (Fig. 4A–D). There are 4 isoforms of p38 MAPK and SB203580 inhibits only two of the 4 isoforms [53]. In order to ascertain role of 2 other forms of p38 MAPK, we knocked down various isoforms of p38 (p38α, p38β, p38γ and p38δ) using siRNA. Among all the isoforms, only p38δ knockdown showed significant decrease in the CCL5 expression. (56.1±5.5% at the level of mRNA and by 43.26±2.21% at the level of protein) (Fig. 4E, F).

### Involvement of C/EBP and AP-1 in CCL5 Expression

**Figure 5:**
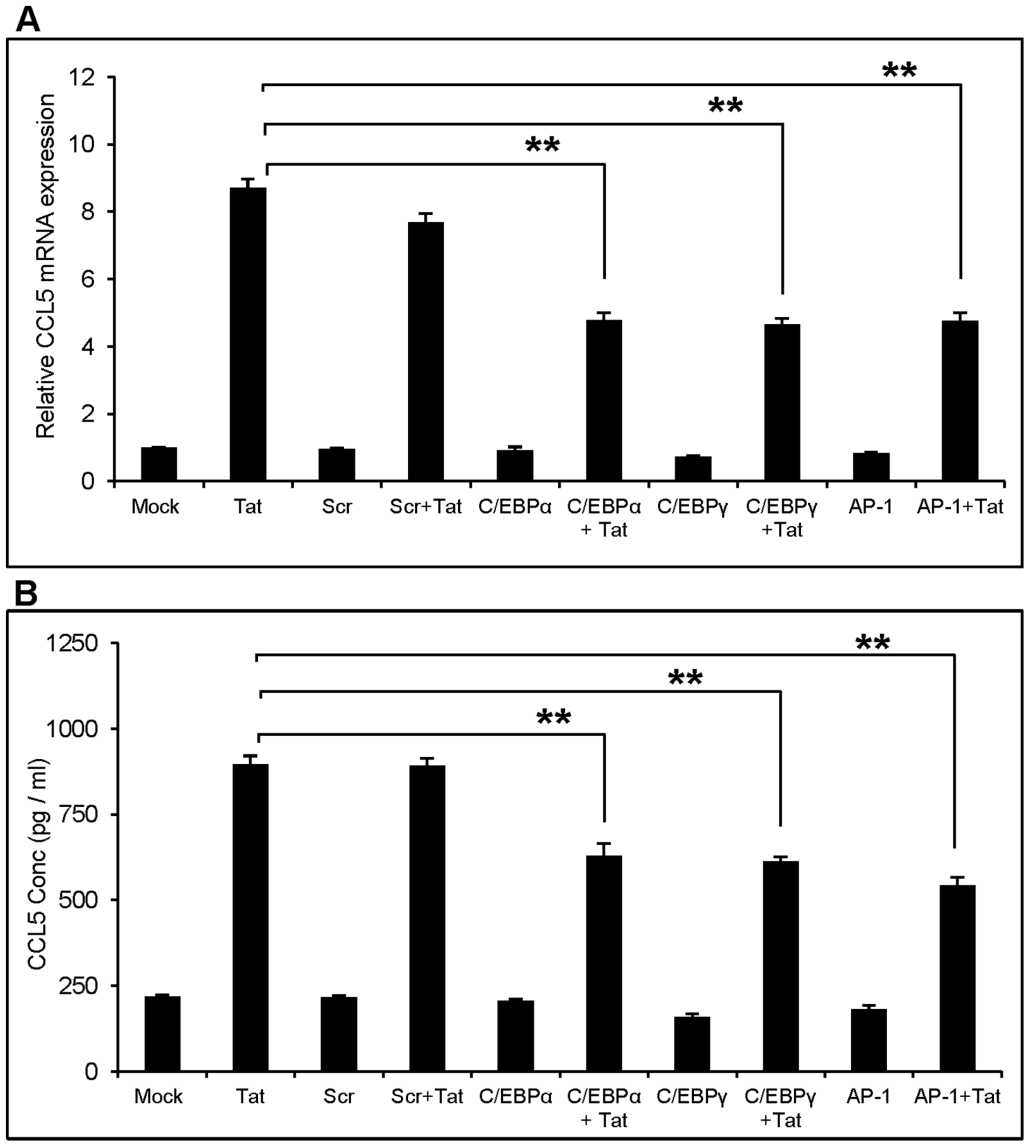
Involvement of C/EBPα, C/EBPγ and AP-1 in HIV-1 Tat mediated induction of CCL5. SVGA astrocytes were transfected with siRNA against C/EBPα, C/EBPγ and AP-1 (A, B) for 48 h before transfecting with HIV-1 Tat plasmid. The expression of CCL5 was measured at mRNA (A) and protein (B) levels. The values represented for mRNA are expressed relative to the mock-transfected controls. Each experiment was performed in triplicate and each bar in the figure represents the mean ± SE of at least three individual experiments. One-way ANOVA was used to perform the statistical analysis and ** denotes p-value ≤0.01.

There are 6 isoforms of CCAT/enhancer binding protein (C/EBP) known in the literature [54] of which p38δ can lead to activation of α and γ isoforms of C/EBP. In addition, p38δ is also known to activate Activator protein-1 (AP-1) [55]. To determine the involvement of these 3 transcription factors, we used siRNA against AP-1, C/EBPα and C/EBPγ, and the CCL5 expression at the mRNA and protein levels were measured 6 h and 48 h post-transfection, respectively. We used scrambled (Scr) siRNA as negative control. In these experiments, the Scr siRNA showed slight decrease in CCL5 expression at RNA level but the change was statistically insignificant. Furthermore, the Scr siRNA did not show any effect on CCL5 expression at protein level suggesting specificity of the effects obtained by using C/EBPα, C/EBPγ and AP-1 siRNA. The C/EBPα knock down declined CCL5 production at mRNA and protein levels by 44.8±4.1% and 30.1±5.9%, respectively. The C/EBPγ and AP-1 also decreased CCL5 production at comparable level. (Fig. 5A, B).

### Role of PI3K/Akt and JAK in HIV-1 Tat Mediated Expression of CCL5

**Figure 6:**
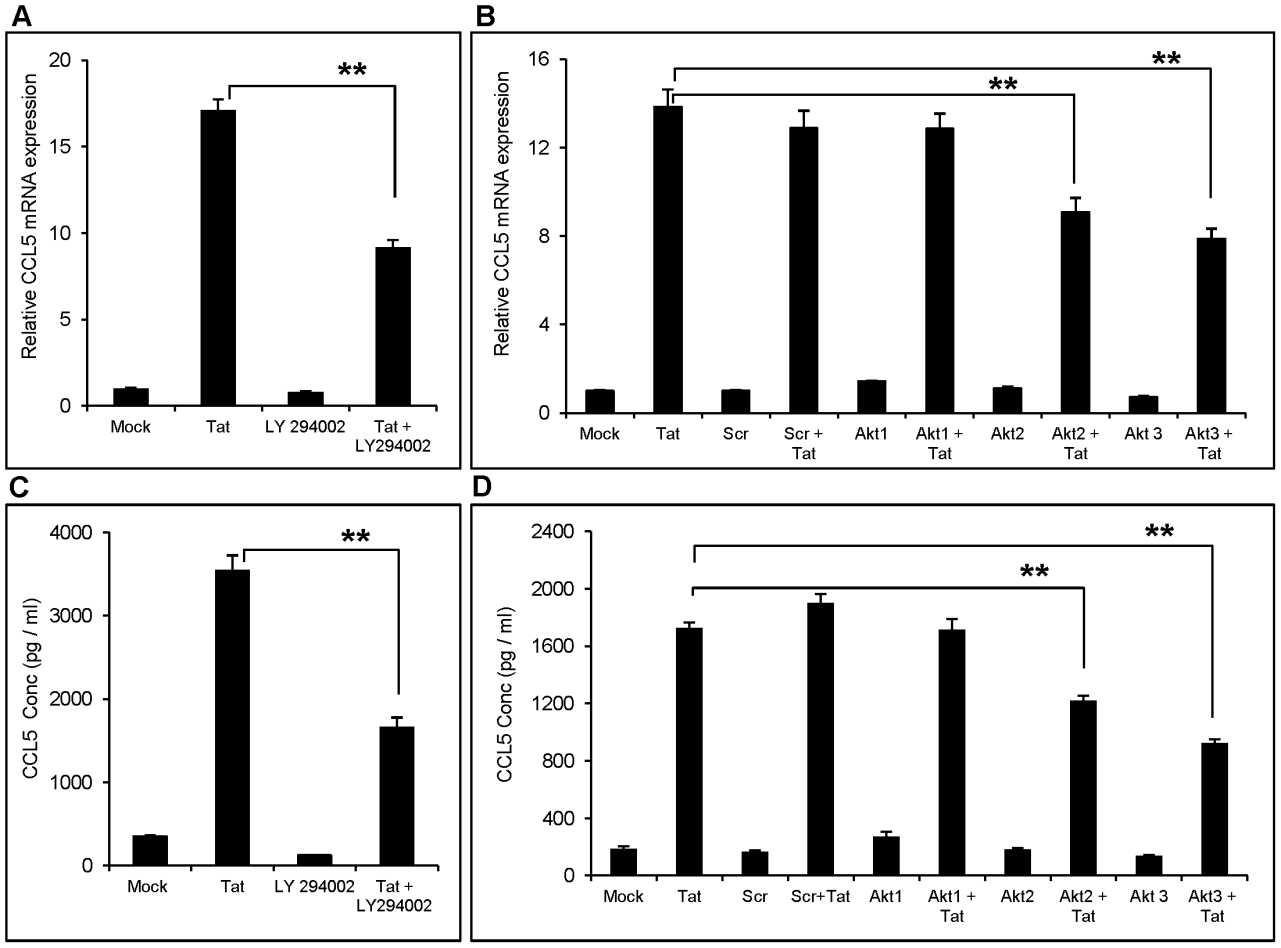
Role of PI3K/Akt in HIV-1 Tat mediated up-regulation of CCL5 in astrocytes. SVGA astrocytes were pretreated with 10 µM of specific PI3K inhibitor (LY294002) (A, B) or transfected with siRNA against different isoforms of Akt (Akt1/2/3) (C, D) prior to transfection with plasmid encoding HIV-1 Tat. Induction of CCL5 was measured at mRNA (A, C) and protein levels (B, D). The values represented for mRNA are expressed relative to the mock-transfected controls. Each experiment was performed in triplicate and each bar in the figure represents the mean ± SE of three individual experiments. One-way ANOVA was used to perform the statistical analysis and ** denotes p-value ≤0.01.

In addition to various MAPK, PI3K/Akt is an alternative signaling mechanism, which activates NF-κB *via* IκB kinase (IKK)-mediated phosphorylation of IκBα [56]. Therefore, we explored the possible involvement of PI3K/Akt in Tat-mediated expression of CCL5. Use of specific Phosphatidylinositide 3-kinase (PI3K) inhibitor, LY294002 decreased CCL5 expression by 46.2±4.3% at mRNA and 53.2±7.44 at protein level (Fig. 6A, B). These results were further confirmed by specific knockdown of various isoforms of protein kinase B (Akt) (Akt1/Akt2/Akt3) using siRNA. The knockdown of Akt2 and Akt3 but not Akt1 reduced the expression of CCL5 by 34.05±7.7% and 42.8% ±6.3% at the level of mRNA and by 29.25±2.86% and 46.4±3.03% at protein levels (Fig. 6C, D).

**Figure 7:**
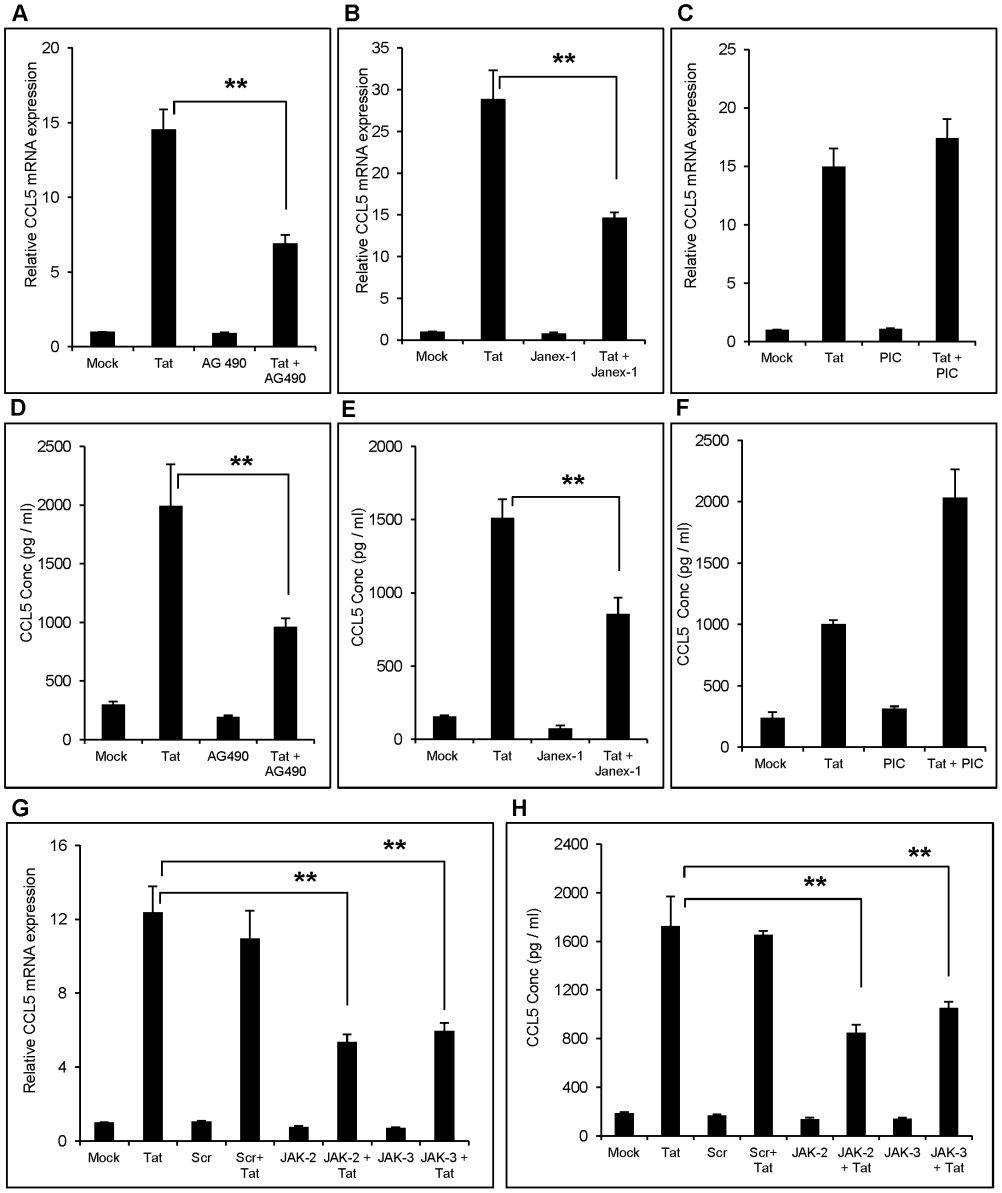
Role of JAK in the induction of CCL5 by HIV-1 Tat in astrocytes. SVGA astrocytes were pretreated with specific 10 µM JAK2 (AG490) (A,B) or 20 µM JAK3 (Janex-1) inhibitors (C, D) or 25 µM JAK1 (E, F) or siRNA to knock down JAK2 and JAK3 (G,H) prior to the transfection with HIV-1 Tat plasmid. The level of CCL5 induction was measured at mRNA (A, C, E, G) and protein levels (B, D, F, H). The values represented for mRNA are expressed relative to the mock-transfected controls. Each experiment was performed in triplicate and each bar in the figure represents the mean ± SE of at least three individual experiments. One-way ANOVA was used to perform the statistical analysis and ** denotes p-value ≤0.01.

Earlier findings by two independent groups have shown a possible connection between Janus kinase (JAK) isoforms and PI3K/Akt. We therefore determined the role of JAK1, JAK2 and JAK3 isoforms in CCL5 regulation. In our study, specific inhibitor for JAK 2 (AG 490) and JAK 3 (Janex-1) but not JAK1 (Picetannol) decreased the expression of CCL5 mRNA by 52.7±8.6% and 49.13±4.7%, respectively, (Fig. 7A, C and E). This effect was also observed at protein level wherein JAK2 and JAK3 specific inhibitors abrogated CCL5 expression by 48.24±7.4% and 43.5±5.1%, respectively (Fig. 7B, D). To our surprise, JAK1 inhibitor induced the protein levels of Tat-mediated CCL5 (Fig. 7F), which seems to be a non-specific effect. The involvement of JAK was further confirmed by knocking down JAK1, JAK2 and JAK3 genes using siRNA. The JAK2 and JAK3 but not JAK1 knockdown significantly reduced CCL5 mRNA expression (56.4±7.4% and 48.3±6.6% for JAK2 and JAK3, respectively) (Fig. 7G). We did not measure effect of JAK1 knockdown on protein expression because it did not show any effect at RNA level. Furthermore, the treatment with JAK1 inhibitor showed increased protein levels of CCL5, which suggested that JAK1 does not play any role in Tat-mediated induction of CCL5. However, both JAK2 and JAK3 knockdown inhibited CCL5 protein production by 50.7±7.4% and 40.4±4.9%, respectively. (Fig. 7 H).

## Discussion

HIV-1 associated neurological deficits range from minor cognitive motor disorders (MCMD) to a more severe form of dementia referred to as HAD. These deficits have been attributed to oxidative stress and pro-inflammatory cytokines among other reasons [20]. Increased levels of pro-inflammatory cytokines/chemokines, including IFN-γ, IL-8 and TNF-α have been reported in not only CSF but also various regions of the brain in individuals infected with HIV-1. [57], [58], [59]. Further, increased CCL5 has not only been detected in the CSF, but it has been directly correlated with viral load in CSF and MCMD [49], [60]. Additionally, HIV-1 proteins (gp120 and Tat) have been shown to increase the CCL5 expression in the cells of CNS origin [35], [38]. The inflammatory response of CCL5 is attributed to its role in recruitment of leucocytes, increase in production of reactive oxygen species and nitric oxide [61], [62] and reduction of anti-inflammatory cytokines such as IL-10 [63]. In this study, we sought to determine the molecular mechanism(s) underlying HIV-1 Tat-mediated CCL5 up-regulation in human astrocytes. We first observed time dependent induction of CCL5 by HIV-1 Tat at both mRNA and protein levels. Our results clearly showed peak CCL5 RNA expression at 1 hour after transfection and, therefore, we believe that CCL5 response is a direct HIV-1 Tat-mediated effect. These results are in agreement with the previous literature reporting the HIV-1 Tat-mediated increase in production of CCL5 in astrocytes [35]. The role of CCL5 in the context of HIV is uncertain. CCL5 interacts with CCR5 receptor to inhibit the replication of various macrophage-tropic strains of HIV-1 at a concentration of 50–250 ng/ml [64], [65]. On the other hand, CCL5 at 1–10 µg/ml concentration increased the HIV-1 infectivity by activating p44/p42 MAPK [66], [67]. CCL5 has also been shown to exhibit neuroprotective properties by decreasing the neuronal apoptosis induced by gp120 [68], [69]. However these concentrations are very high when compared to the concentrations that have been reported in the CSF of HIV infected patients suffering from dementia and opportunistic infections where CCL5 concentration have been reported to be in the range of 50–200 pg/ml [49], [70]. In an another case of *Neisseria meningitides/*meningioma cell culture system, 5–10 ng/ml concentrations of CCL5 were shown to play a role in inflammatory responses [71]. These studies suggest that, a lower concentration of CCL5 perhaps causes inflammation whereas higher concentration might be involved in protective effect. In our study, we achieved the peak concentration of CCL5 in the range of 2–4 ng/ml, which would be expected to lead into pro-inflammatory response.

NF-κB plays a critical role in regulation of various cytokines/chemokines during inflammatory responses owing to the presence of binding site for NF-κB in their promoter regions. Previous reports indicated the role of HIV-1 Tat in the activation of NF-κB in primary human astrocytes [72]. NF-κB has also been shown to be associated with gp120-mediated increase in CCL5 production in astrocytes [38]. In order to determine involvement of NF-κB, we used SC514, a specific inhibitor of IκB kinase 2 (IKK2). SC514 prevents the degradation of IκBα and thereby prevents the translocation of NF-κB [45]. In accordance with the previous reports, we also observed SC514 mediated reduction of CCL5 expression suggesting a role for NF-κB. Our results also suggested that both p50 and p65 subunits are involved in this process as knockdown of both p50 and p65 resulted in reduction of the CCL5 production, confirming our results with the chemical antagonist. These observations clearly suggest the involvement of NF-κB pathway in the signaling mechanism underlying Tat-mediated CCL5 production in astrocytes.

Various MAPKs are known to play an important role in the up-regulation of many cytokines by activating NF-κB [52], [73]. Particularly, p38 MAPK, which belongs to the family of serine/threonine phospho-kinases, is known to be involved in the regulation of variety of cytokines/chemokines [74], [75]. p38 MAPK consists of four different isoforms (α/β/γ/δ), of which p38α and p38β lead to the activation of NF-κB [76]. In order to study the role of p38 MAPK, we first employed a specific chemical antagonist, SB203580, that blocks p38α and p38β isoforms but not p38γ and p38δ isoforms [53]. To our surprise, pretreatment with SB203580 did not affect the Tat-mediated expression of CCL5 at both mRNA as well as protein levels. These results clearly indicate that p38 MAPK is not involved in NF-κB activation. In order to further dissect the role of other isoforms of p38, we individually knocked them down using siRNA. Among all, siRNA against p38δ significantly decreased the expression of CCL5. Since p38δ does not lead to the activation of NF-κB, we investigated the involvement of other transcription factors such as, AP-1, C/EBPα and C/EBPγ, which can bind to the promoter of CCL5 [77], [78]. Our results showed reduction of CCL5 levels after knocking down AP-1, C/EBPα and C/EBPγ which clearly suggests the involvement of these transcription factors in the regulation of HIV-1 Tat-mediated CCL5 expression.

We further dissected the signaling upstream of NF-κB as p38 MAPK was found not to be involved NF-κB activation. In addition to various MAPKs, PI3K/Akt also serves as a regulator, upstream of NF-κB [79]. It can phosphorylate IKBα and thereby promote the translocation of NF-κB into the nucleus. In our study, we found that pre-treatment of astrocytes with PI3K inhibitor, LY294002 decreased the HIV-1 Tat-mediated expression of CCL5. Akt belongs to the family of serine/threonine kinases, and exists in three different isoforms (Akt1/PKBα, Akt2/PKBβ, Akt3/PKBγ). These isoforms mainly differ in their phosphorylation sites and in their tissue distributions and physiological functions [80]. In addition, Akt3 is predominantly important in the brain since it contributes for more than 50% of all the Akt isoforms found in the brain [81]. In our study, siRNA against Akt2 and Akt3, but not Akt1 showed substantial reduction in the expression of CCL5. These findings suggest that perhaps the brain specific isoforms of Akt; i.e. Akt2 and Akt3, play an important role in the expression of CCL5, *via* activation of NF-κB, which serves as one of the several transcription factor in this process.

JAK/STAT pathway is a major cytokine-signaling mechanism, which is known to activate PI3K/Akt in addition to various Signal Transducer and Activator of Transcription (STAT). In addition, the chemical inhibitors of both the JAK2 and JAK3 reduced IL-6 and Toll like receptor-mediated phosphorylation of Akt, suggesting a link between JAK and PI3K/Akt pathway [82], [83]. Our results with the specific inhibitors for JAK2 (AG490) and JAK3 (Janex-1) suggested a similar phenomenon in Tat-mediated CCL5 expressions, wherein Tat may activate JAK, which can further lead to activation of PI3K/Akt. Furthermore, knock down of JAK2 and JAK3 isoforms by siRNA also resulted in the decreased expression of CCL5 by HIV-1 Tat confirming the involvement of JAKs in Tat-mediated CCL5 expression.

## Conclusions

**Figure 8:**
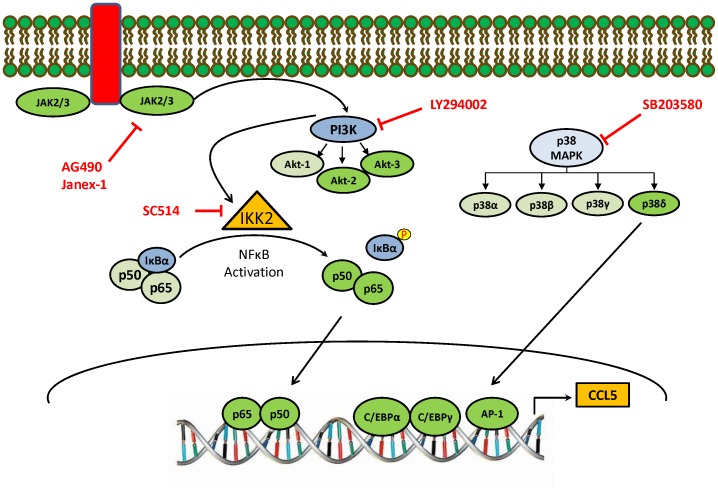
Schematic representation of the signaling pathways involved in HIV-1 Tat mediated up-regulation of CCL5 in astrocytes. The induction of CCL5 by HIV-1 Tat involved JAK/PI3K/Akt and p38 MAP kinase pathways. These signaling pathways differentially regulated the induction of CCL5 by activating various transcription factors, including NF-κB, C/EBPα, C/EBPγ and AP-1. The target molecules of siRNA are indicated in green color and the involvement of a specific isoform is shown in brighter color and the absence is shown in pale color. The specific inhibitors for their respective targets are shown in red.

In summary, our results clearly demonstrate that HIV-1 Tat increased the expression of CCL5 from astrocytes in a time dependent manner. In view of our findings, the up-regulation of CCL5 in astrocytes by HIV-1 Tat involves different signaling pathways, including JAK/PI3K/Akt and p38 MAPK pathways. The activation of JAK isoforms, JAK2 and JAK3 may lead to the activation of Akt2 and Akt3, which in turn can activate NF-κB. This phenomenon was isoform specific as JAK1 and Akt1 were not involved in the CCL5 up-regulation. Similarly, among various isoforms of p38, only p38δ was involved, which in turn increased the CCL5 expression *via* C/EBPα, C/EBPγ and AP-1 transcription factors. Together, all these findings indicate that the Tat-mediated CCL5 expression involves multifaceted signaling mechanisms in isoform specific manner (Fig. 8).
